# GM-CSF in murine psoriasiform dermatitis: Redundant and pathogenic roles uncovered by antibody-induced neutralization and genetic deficiency

**DOI:** 10.1371/journal.pone.0182646

**Published:** 2017-08-04

**Authors:** Tatjana Scholz, Andreas Weigert, Bernhard Brüne, Christian D. Sadik, Beate Böhm, Harald Burkhardt

**Affiliations:** 1 Fraunhofer Institute for Molecular Biology and Applied Ecology IME, Project Group Translational Medicine & Pharmacology TMP, Goethe University, Frankfurt am Main, Germany; 2 Institute of Biochemistry I-Pathobiochemistry, Faculty of Medicine, Goethe University, Frankfurt am Main, Germany; 3 Department of Dermatology, Allergy, and Venereology, University of Lübeck, Lübeck, Germany; 4 Division of Rheumatology, University Hospital Frankfurt, Goethe University, Frankfurt am Main, Germany; Cincinnati Children's Hospital Medical Center, UNITED STATES

## Abstract

Granulocyte-macrophage colony-stimulating factor (GM-CSF) is a pleiotropic, Th17-derived cytokine thought to critically contribute to the pathogenesis of diverse autoimmune diseases, including rheumatoid arthritis and psoriasis. Treatment with monoclonal antibodies that block GM-CSF activity is associated with favorable therapeutic effects in patients with rheumatoid arthritis. We evaluated the role of GM-CSF as a potential target for therapeutic interference in psoriasis using a combined pharmacologic and genetic approach and the mouse model of imiquimod-induced psoriasiform dermatitis (IMQPD). Neutralization of murine GM-CSF by an anti-GM-CSF antibody ameliorated IMQPD. In contrast, genetic deficiency in GM-CSF did not alter the course of IMQPD, suggesting the existence of mechanisms compensating for chronic, but not acute, absence of GM-CSF. Further investigation uncovered an alternative pathogenic pathway for IMQPD in the absence of GM-CSF characterized by an expanded plasmacytoid dendritic cell population and release of IFNα and IL-22. This pathway was not activated in wild-type mice during short-term anti-GM-CSF treatment. Our investigations support the potential value of GM-CSF as a therapeutic target in psoriatic disease. The discovery of an alternative pathogenic pathway for psoriasiform dermatitis in the permanent absence of GM-CSF, however, suggests the need for monitoring during therapeutic use of long-term GM-CSF blockade.

## Introduction

Psoriasis is a complex chronic inflammatory disease of the skin featuring keratinocyte hyperproliferation and dysregulation of terminal keratinocyte differentiation, resulting in a thickening of the epidermis (acanthosis) and a marked prolongation of the rete ridges (papillomatosis). In parallel, there is a pronounced infiltration of the skin by diverse types of immune cells, including CD4^+^ and CD8^+^ T lymphocytes, neutrophils, macrophages, dendritic cells (DCs), and mast cells [1]. In the last decade, the IL-23/IL-17 pathway has been highlighted as an essential driver of psoriasis; treatment regimens therapeutically inhibiting this pathway by IL-23- or IL-17-blocking antibodies have proven highly effective in clinical use [2–4]. According to the current model, antigen-activated T-helper type-17 (Th17) lymphocytes acquire the capability to produce IL-17A by interaction with DCs that promote Th17 differentiation and the release of cytokines, including IL-23 [5, 6]. The activation of the IL-23/Th17 axis subsequently triggers the release of proinflammatory mediators, including IL-22 and granulocyte-macrophage colony-stimulating factor (GM-CSF) [2, 7, 8].

GM-CSF is a proinflammatory cytokine and myelopoietic differentiation factor involved in macrophage activation towards a proinflammatory phenotype [8], which is characterized by an enhanced IL-6 and TNFα response pattern [9]. The inhibition of GM-CSF by recombinant antibodies directed to GM-CSF itself [10] or its receptor [11] has recently been shown to ameliorate rheumatoid arthritis. Several lines of evidence also suggest a role for GM-CSF in the pathogenesis of psoriasis. In particular, the therapeutic application of recombinant GM-CSF has been reported to result in the emergence of new onset [12] and re-exacerbated psoriatic disease [13]. The therapeutic potential of GM-CSF inhibition in psoriasis is currently being tested in a phase II clinical trial examining the effect of the GM-CSF-neutralizing antibody namilumab in psoriasis patients (ClinicalTrials.gov NCT02129777).

The present investigation was performed to evaluate the therapeutic potential of GM-CSF blockade in the treatment of psoriasis by assessing the effect of an anti-murine GM-CSF monoclonal antibody (mAb) in the imiquimod (IMQ)-induced psoriasiform dermatitis (IMQPD) mouse model of plaque psoriasis. In this model, dermatitis that exhibits features similar to those of psoriasis is induced by daily application of IMQ on the shaved mouse back skin, thus provoking the development of an IL-23/IL-17-dependent dermal inflammation with scaly skin lesions resembling plaque-type psoriasis [14]. Although GM-CSF neutralization proved effective in ameliorating psoriasiform dermatitis, mice genetically deficient in GM-CSF surprisingly developed IMQPD that was as severe as that observed in wild-type controls. Our subsequent mechanistic studies uncovered an alternative pathogenic pathway driven by IFNα and IL-22 that was activated under conditions of chronic deficiency in GM-CSF. The existence of this alternative pathway warrants caution for the longterm use of GM-CSF inhibitors in the treatment of chronic inflammatory diseases, particularly in psoriasis.

## Materials and methods

### Mice

Experimental protocols were approved by the Hessian Animal Care and Use Committee (approval numbers F144/11 and FK/1048), and animal study methods were carried out in accordance with the relevant guidelines and regulations from this committee. Male GM-CSF^-/-^ mice in a C57Bl/6J background were kindly provided by Prof. R. Ludwig (Department of Dermatology; University of Lübeck) and were established as previously described [15]. Age-matched male wild-type (GM-CSF-sufficient) C57Bl/6J mice were used as control animals. All animals were bred in our home facility. Mice were maintained in a temperature-controlled environment with a 12-hour light/12-hour dark cycle and were administered standard laboratory food and water *ad libitum*. To minimize animal distress, mice were provided with HydroGel^®^ (ClearH_2_O, Westbrook, ME, USA). During induction of psoriasiform dermatitis, humane endpoint criteria for immediate euthanization were set in regard to changes in body weight, external physical appearance, and behavior; animals were monitored daily. No animal matched these endpoint criteria so premature euthanization was not required. For euthanasia at the study endpoint, animals were anesthetized by inhalation of isoflurane and sacrificed by cervical dislocation.

### IMQPD

Psoriasiform dermatitis was induced by IMQ as described previously [14]. For GM-CSF-blocking treatment, a purified recombinant GM-CSF-neutralizing IgG2a monoclonal antibody (MOR012507), originally selected from a murine phage library (MorphoSys, Martinsried, Germany), was used. MOR012507 was tested for its capacity to neutralize murine GM-CSF by growing 3x10^4^ cells/well of GM-CSF-dependent FDCP-1 cells in the presence of 0.25 ng/mL mouse GM-CSF and different concentrations of MOR012507 for 72 h. The GM-CSF-neutralizing potential of MOR012507 was determined in a bioassay measuring FDCP-1 cell viability through the addition of XTT reagent (Roche Diagnostics, Indianapolis, IN); 90% inhibition was achieved at 5.8 ± 0.2 μg of MOR012507. For the treatment protocol, an antibody dose was calculated to achieve an approximately 10X higher trough serum concentration than the previously determined inhibitory dose. Accordingly, 350 μg of antibody per mouse (MOR12507 [verum] or IgG2a [isotype control; Biolegends, San Diego, CA]) was injected intraperitoneally on day 0, 3 and 5 of IMQPD. Skin manifestations were assessed using clinical scoring of erythema, scaling, and thickness to derive a composite PASI score [14] by an investigator blinded for treatment groups.

### Sample preparation for single-cell staining and *in vitro* experiments

Fat and connective tissue were removed from skin tissue and the skin was cut into small pieces, added to RPMI 1640, 0.5 mg/ml liberase, and 0.5 mg/ml DNase I, and incubated for 1.5 h at 37°C. Digestion was stopped by addition of RPMI 1640 plus 10% FCS. Tissue was processed for 7 min in a 50 μm BD^™^ Medimachine Medicon (BD Biosciences, Heidelberg, Germany). Cells were washed with 2 x 10 ml RPMI 1640 plus 10% FCS. Cells from skin, spleen, and lymph nodes were isolated using a 70 μm cell strainer (Greiner, Bio-One GmbH, Frickenhausen, Germany). Erythrocytes in the spleen were lysed.

### Flow cytometry

Fc receptor binding was blocked by CD16/CD32 anti-mouse antibody (Miltenyi Biotec, Bergisch Gladbach, Germany) for 15 min on ice followed by 30 min incubation in the dark with fluorochrome-labeled antibodies for specific cell markers (S1 Table). To determine absolute cell numbers for evaluation of pDC distribution in lymphoid organs, counting beads (Bangs Laboratories, Fishers, IN) were used and counted cells were normalized to bead input. The samples were measured on a LSRII Fortessa flow cytometer (BD Biosciences) and assessed by FlowJo software (Treestar, Ashland, OR).

### Immunohistochemistry

Samples were fixed in 4% PFA for 4 h, dehydrated in an ascending alcohol series (Sigma-Aldrich, Hamburg, Germany), embedded, and sectioned. Hematoxylin and eosin staining was performed, and splenic pDC were identified by the Siglec-H surface marker (clone 23M15C8; Novus Biologicals, Littleton, CO) using the Dual Endogenous Enzyme Block and Liquid Permanent Red (Dako Deutschland GmbH, Hamburg, Germany), as described by the manufacturer, and conjugated anti-rat antibody (Promega, Mannheim, Germany). Neutrophils were stained by immunofluorescence using anti-mouse Ly-6B.2 (clone 7/4) (Bio-Rad AbD Serotec GmbH, Puchheim, Germany) and anti-rat IgG Cross Adsorbed Secondary Antibody, DyLight 594 conjugate (Thermo Scientific, Karlsruhe, Germany), and embedded in ProLong^®^ Gold Antifade Mountant with DAPI (Thermo Scientific). Analyses were done using an AxioVision 4.8 (Zeiss, Oberkochen, Germany).

### RNA isolation and quantitative PCR (qPCR)

For RNA isolation, snap frozen skin tissue was dissected in peqGold RNAPure^™^ (Peqlab, Erlangen, Germany) by IKA^®^T25 digital UltraTurrax^®^ (IKA^®^-Werke GmbH & Co KN, Staufen, Germany) and RNA preparation was performed as described by the manufacturer. cDNA synthesis from 1 μg RNA was run according to the Maxima First Strand cDNA Synthesis Kit for RT-qPCR (Thermo Scientific). qPCR was performed as described previously [16]; actin was used as an internal control. Primer sequences used to detect cytokine mRNAs are listed in S2 Table.

### T cell stimulation

Splenocytes were isolated as single cell suspensions and T cells were activated by antibody-induced stimulation of CD3 and CD28 for three days (anti-CD3, clone 145-2C11; anti-CD28, clone 37.51) (Biolegends). Supernatants were subsequently analyzed for cytokine release.

### Cytokine measurements

IL-22 was quantified by mouse IL-22 ELISA Ready-SET-Go!^®^ (eBioscience Frankfurt, Germany) according to the manufacturer’s instructions. IL-17 was determined by cytometric bead array (BD Bioscience) as described by the manufacturer.

### Statistical analyses

All statistical analysis tests were performed using Graphpad Prism 5 software. The Mann-Whitney-U-Test was used to determine the statistical significance between data from the control groups and anti-GM-CSF-treated mice and between data rom wild-type and GM-CSF^-/-^ mice. P values < 0.05 were considered statistically significant.

## Results

### GM-CSF neutralization ameliorates IMQPD in wild-type mice

**Fig 1:**
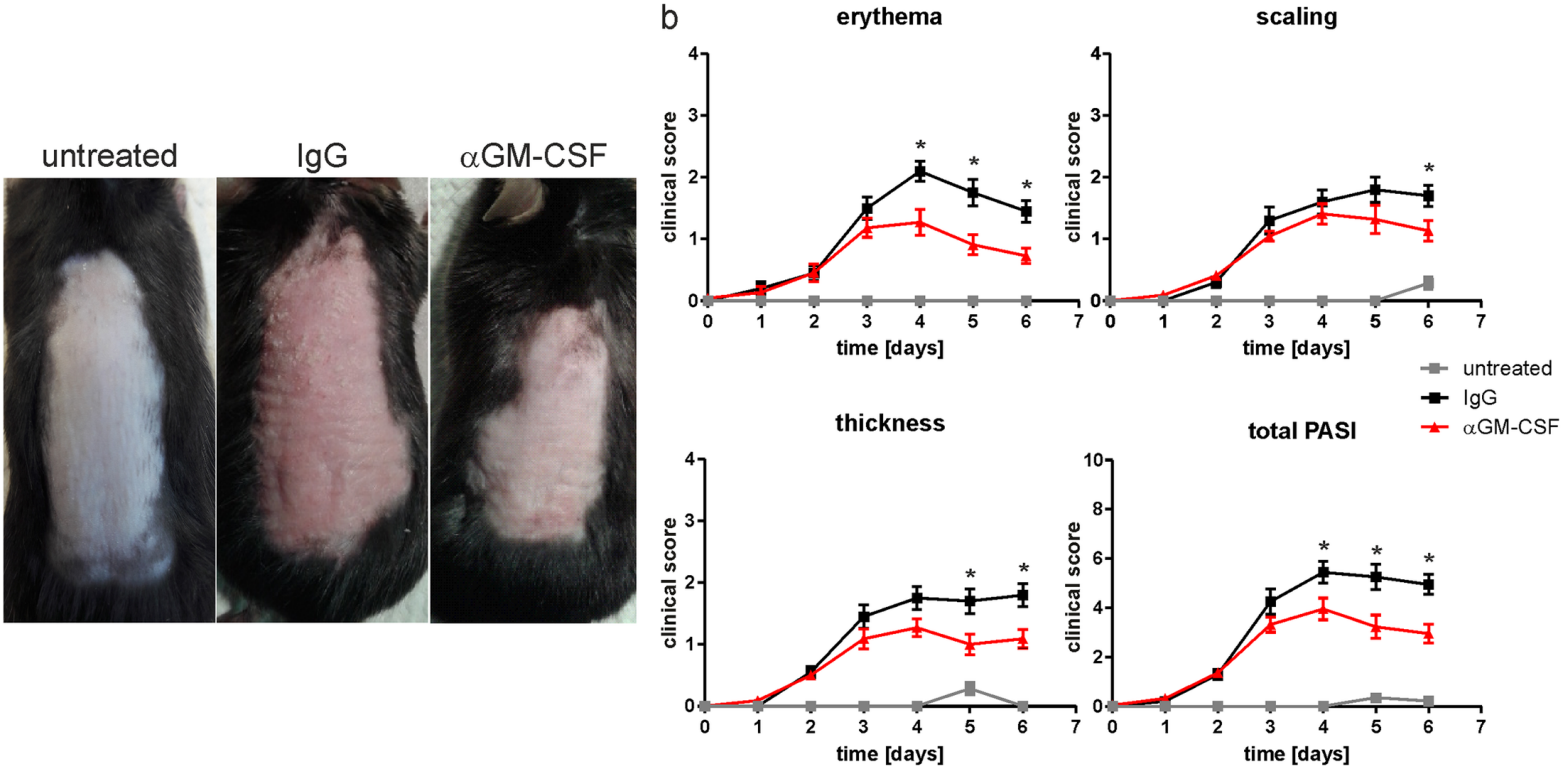
Neutralization of GM-CSF reduces the clinical parameters of IMQ-induced psoriasis. (a) Mouse back skin in mice with IMQPD. Psoriasis was induced by topical application of IMQ in wild-type mice treated with isotype control (IgG n = 10) or a GM-CSF neutralizing antibody (αGM-CSF n = 11) with mice who were not treated with IMQ as comparison (untreated n = 7); mice were sacrificed on day 6. (b) Time course of erythema, scaling, stiffness, and total PASI scores in treated and untreated mice. Graphs show mean values ± SEM. *p-value<0.05 αGM-CSF-treated mice compared with IgG control group as determined by Mann-Whitney-U-Test.

**Fig 2:**
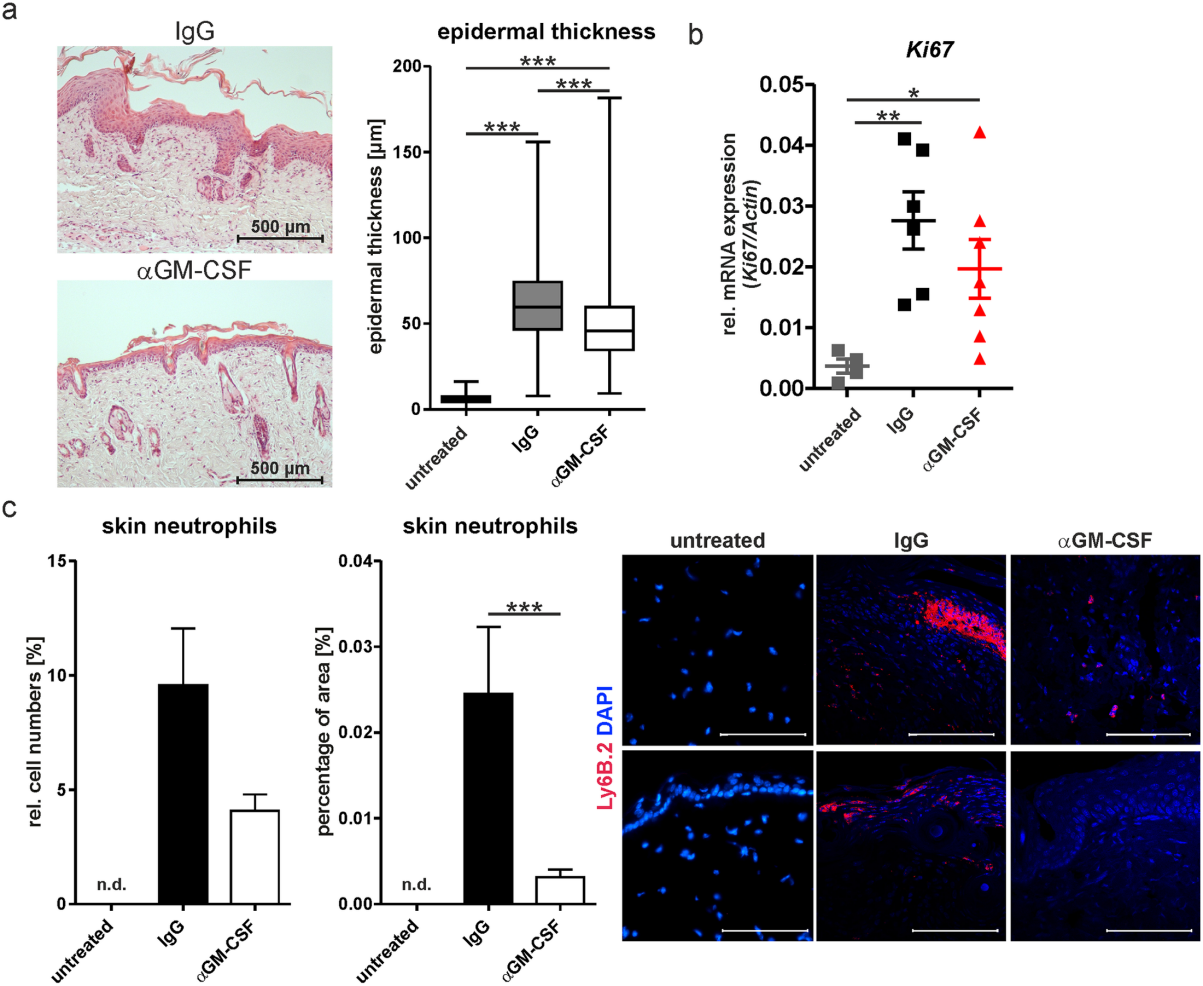
Neutralization of GM-CSF ameliorates cellular changes associated with IMQ-induced psoriasis. Psoriasis was induced by topical application of IMQ in wild-type mice treated with isotype control (IgG) or a GM-CSF neutralizing antibody (αGM-CSF); mice were sacrificed on day 6. Untreated animals were not treated with IMQ. (a) Representative hematoxylin and eosin (HE) staining of back skin and quantification of epidermal thickness of HE-stained samples (untreated n = 3; IgG n = 5; αGM-CSF n = 6). (b) *Ki67* mRNA expression (right: untreated n = 4; IgG n = 6; αGM-CSF n = 7; each symbol represents the quantified measured mRNA of one mouse) in skin by qPCR. (c) Quantification of neutrophil distribution by flow cytometry (left: n = 4) and by immunofluorescence staining with Ly6B.2 (middle: untreated n = 4; IgG n = 8; αGM-CSF n = 11) based on representative staining patterns (right). Neutrophils were not detectable (n.d.) in untreated mice. Scale bars equal 100 μm. Graphs show mean values ± SEM, except for right panel of (a) (Box-Whisker-Plot of epidermal thickness). *p-value<0.05, **p-value<0.01, ***p-value< 0.001 as determined by Mann-Whitney-U-Test.

In order to assess the potential role of GM-CSF as a therapeutic target in psoriatic skin disease, we tested the effect of injections of a GM-CSF-neutralizing recombinant mAb, MOR012507, on development of IMQPD. MOR012507 ameliorated clinical severity of IMQPD (Fig 1a) by approximately 30% compared with the IgG control, as evaluated by the Psoriasis Area and Severity Index (PASI) score and its components at day 6 (Fig 1b). Decreases in PASI were also mirrored by significant reductions in epidermal thickness compared with the IgG control (Fig 2a). IMQ increased mRNA expression of the proliferation marker *Ki67* compared with untreated mice (Fig 2b), but there was no difference between the αGM-CSF- and IgG-treated mice. Recruitment of neutrophils into the skin following IMQ treatment was reduced by GM-CSF inhibition (Fig 2c); a statistically significant difference was observed for the percentage of area occupied by skin neutrophils as assessed by immunofluorescent assays with a neutrophil-specific marker, although not for their relative cell numbers as assessed by flow cytometry. As expected, healthy mice that had not undergone IMQ treatment did not show an increase in epidermal thickness or *Ki67* expression (Fig 2a and 2b).

### Genetic deficiency in GM-CSF does not protect against IMQPD

Given the effectiveness of MOR012507 in reducing the severity of IMQPD, we investigated the impact of GM-CSF deficiency on the course of IMQPD in C57Bl/6J mice with a genetically engineered disruption of the gene encoding GM-CSF, *Csf2*. The absence of GM-CSF was confirmed through qPCR analysis of *Csf2* gene expression. Quantifiable levels of *Csf2* were not detected in the GM-CSF-deficient mice (data not shown).

**Fig 3:**
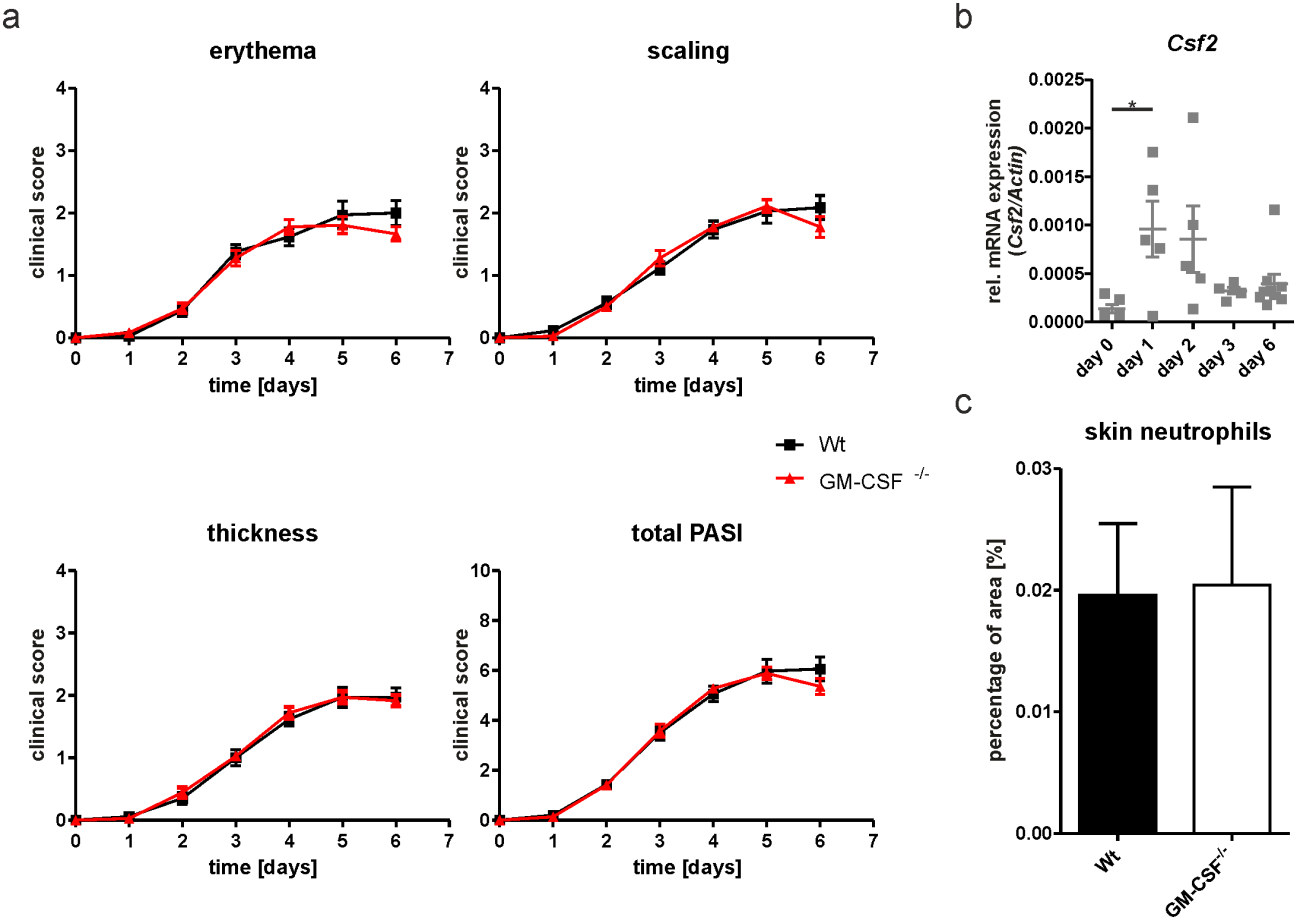
GM-CSF deficiency does not protect mice from IMQPD. (a) Time course of erythema, scaling, stiffness and total PASI scores in IMQ-treated wild-type (Wt n = 12) and GM-CSF^-/-^ (n = 13) mice. (b) *Csf2* mRNA expression relative to actin mRNA in skin of wild-type mice (days 1–3 n = 5; day 6 n = 9) following IMQ treatment. (c) Neutrophil distribution in skin at day 6 after IMQ application by immunofluorescence staining Ly6B.2 (Wt n = 6; GM-CSF^-/-^ n = 5). Graphs show mean values ± SEM. *p-value<0.05.

**Fig 4:**
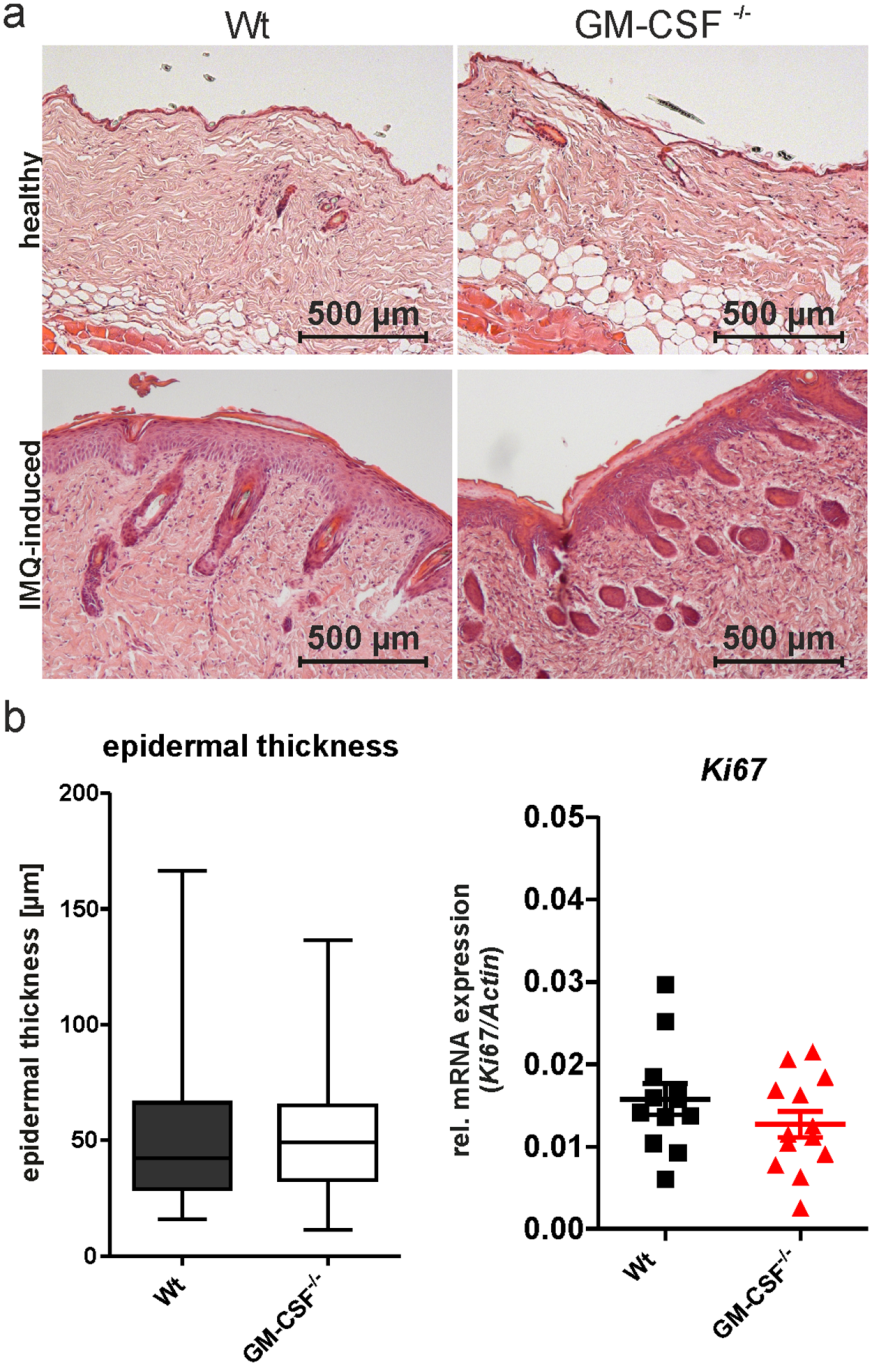
GM-CSF deficiency does not alter epidermal thickening during IMQPD. Mice were sacrificed at day 6 after IMQ application. (a) Representative hematoxylin and eosin (HE) staining of healthy (upper panel) and inflamed skin following IMQ application (bottom panel) in wild-type (Wt) and GM-CSF^-/-^ mice. (b) Quantification of epidermal thickness of HE-stained histological samples (left: Wt n = 5; GM-CSF^-/-^ -n = 4) and *Ki67* mRNA expression in skin by qPCR (right: Wt n = 12; GM-CSF^-/-^ n = 13; each symbol represents the quantified measured mRNA of one mouse). Scale bar as indicated.

Surprisingly, genetic GM-CSF deficiency (GM-CSF^-/-^) did not affect the development of IMQPD (Fig 3a), even though expression of the *Csf2* gene in the skin is an early event during IMQPD in wild-type mice (Fig 3b). Nevertheless, GM-CSF^-/-^ and wild-type mice showed similar time courses for PASI total and component scores following application of IMQ (Fig 3a). Similarly, there were no differences in neutrophil infiltration (Fig 3c), epidermal thickness (Fig 4a and 4b), or *Ki67* expression (Fig 4b) between GM-CSF^-/-^ mice and wild-type controls. These findings demonstrated that, in sharp contrast to acute GM-CSF inhibition by neutralizing antibody, genetic deficiency in GM-CSF does not counteract the development of IMQPD. These divergent results may conceivably be due to the activation of alternative proinflammatory pathways in GM-CSF^-/-^ mice that substitute for GM-CSF and allow the development of full-blown skin disease.

### Differences in cytokine signatures following IMQPD induction in GM-CSF^-/-^ and wild-type mice suggest alternative pathogenic pathways

**Fig 5:**
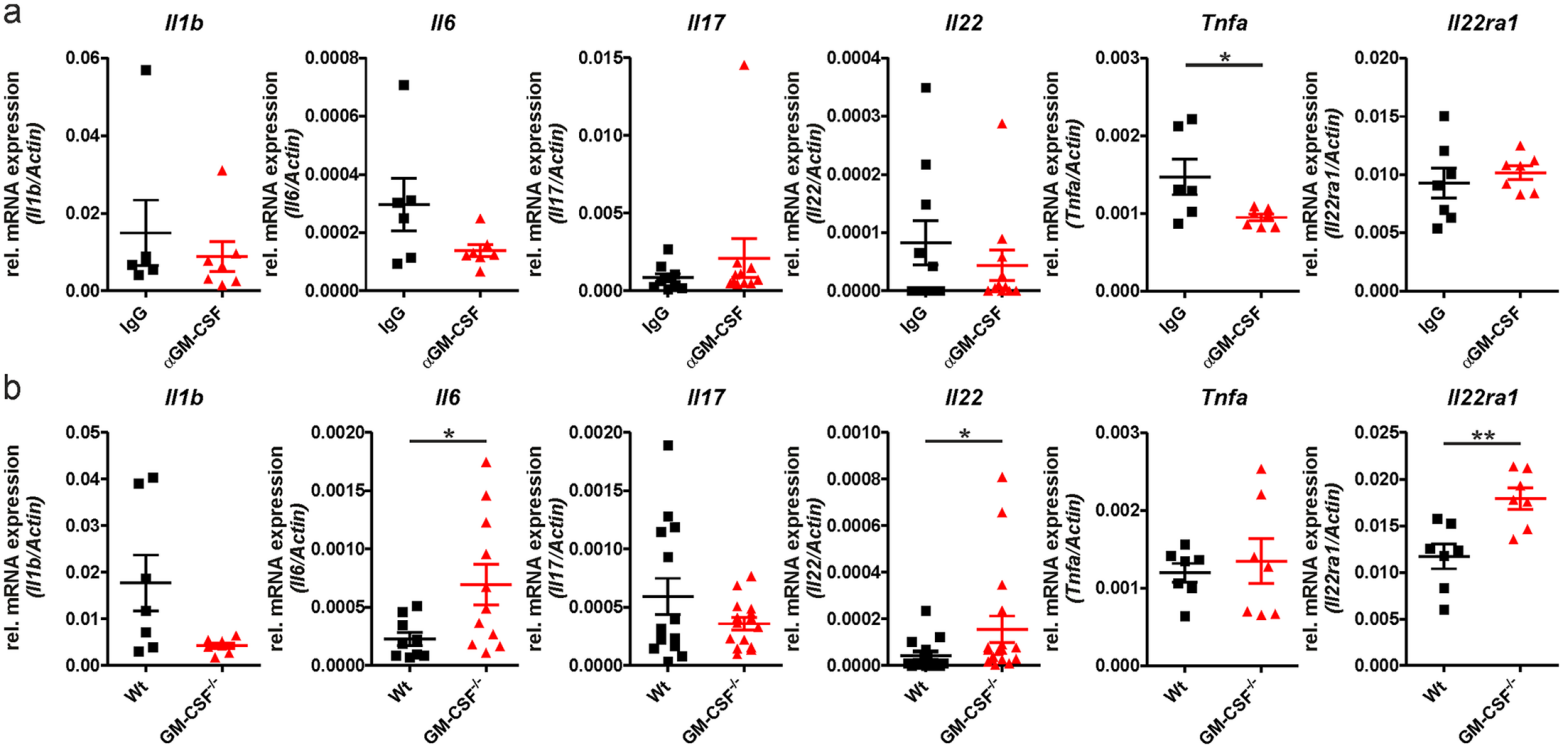
Anti-GM-CSF treatment leads to reduced mRNA expression of pro-inflammatory mediators at day 6 that are not observed with genetic GM-CSF deficiency. (a) Il1b, Il6, Il17, Il22, Tnfa, and Il22ra1 mRNA levels relative to actin mRNA in IgG isotype-treated (n = at least 6) and anti-GM-CSF-treated (n = at least 7) wild-type mice; each symbol represents the quantified measured mRNA of one mouse. IMQPD was induced in mice by topical application of IMQ, and mRNA expression was analyzed in skin cells at day 6. (b) mRNA levels relative to actin mRNA in wild-type (Wt) and GM-CSF^-/-^ mice (n = at least 7 for each). Symbols in graphs indicate mRNA levels in individual mice; horizontal bars show mean values ± SEM. *p-value<0.05, **p-value< 0.01, compared with control group as determined by the Mann-Whitney-U-Test.

In order to test the hypothesis that additional pathogenic pathways become active in IMQPD in GM-CSF^-/-^ *vs*. wild-type mice, we determined the mRNA expression levels of relevant proinflammatory cytokines (IL-1β, IL-6, TNFα, IL-17, IL-22, and the alpha subunit of its receptor IL-22RA1) in the skin at day 6 after disease induction. Cytokine levels were evaluated in wild-type mice treated with either MOR012507 or an IgG control and in GM-CSF^-/-^ mice. In wild-type mice (Fig 5a), treatment with the GM-CSF-neutralizing antibody MOR012507 was associated with numeric reductions in cytokine mRNA levels of IL-1β, IL-6, TNFα, and IL-22 compared with isotype-treated controls, although a statistically significant difference was only reached for TNFα. The mRNA levels of IL-17 and IL-22RA1 remained unaffected by MOR012507. We further observed a statistically significant increase in mRNA levels for IL-6, IL-22, and IL-22RA1 in GM-CSF^-/-^ mice compared with wild-type mice following IMQPD induction (Fig 5b). Other cytokines did not significantly differ. These findings suggest that in GM-CSF^-/-^ mice, but not wild-type, an alternative pathway involving IL-6 and IL-22 that significantly contributes to skin inflammation is activated upon IMQ application.

### Plasmacytoid dendritic cell (pDC) cell counts are enhanced in GM-CSF^-/-^ mice

**Fig 6:**
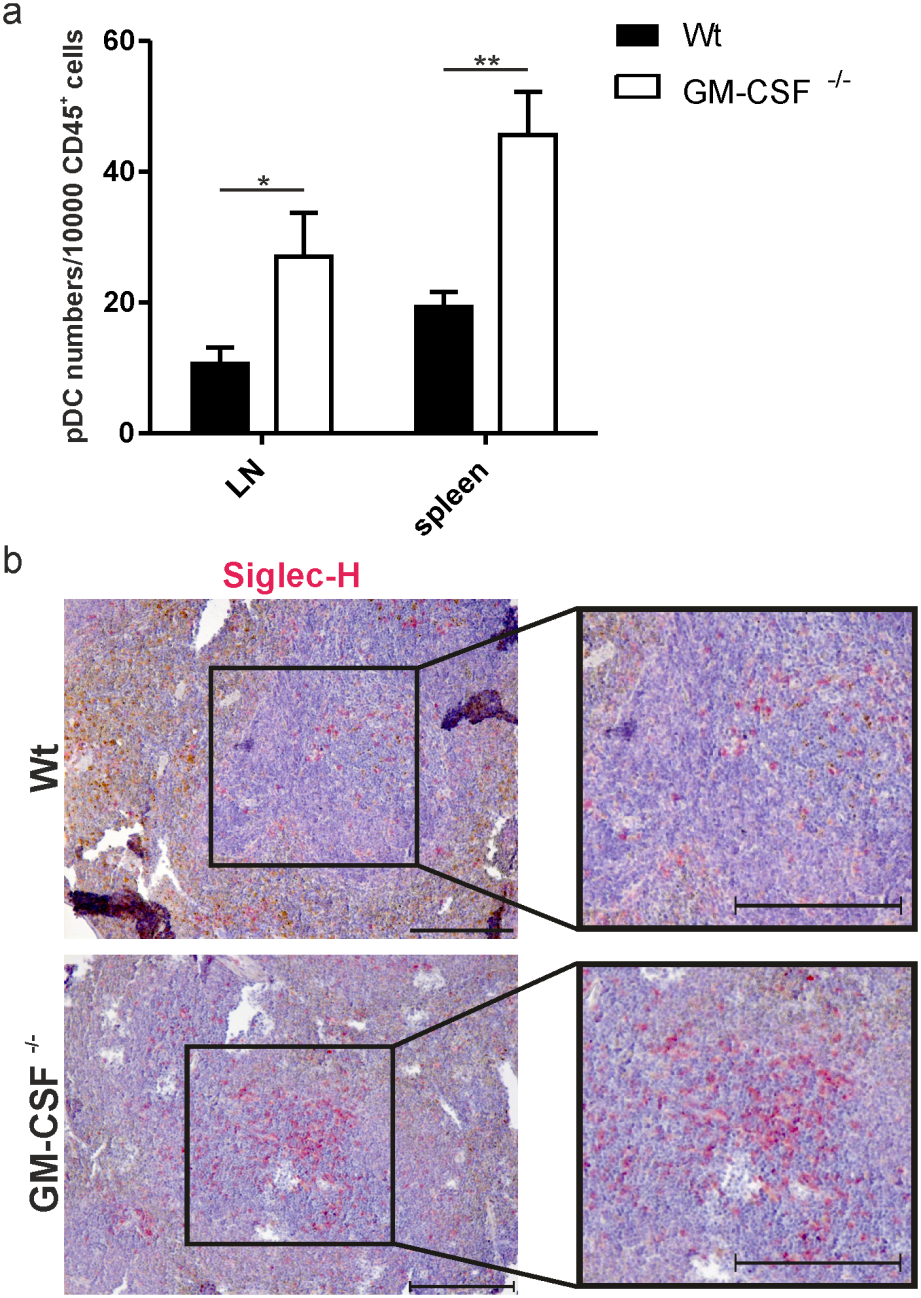
GM-CSF deficiency enhances the pDC compartment. (a) Quantification of pDC in skin draining lymph nodes (LN) and spleen (CD45^+^Siglec-H^+^, CD11cint) in healthy wild-type (Wt n = 4) and GM-CSF^-/-^ (n = 4) mice without prior IMQ application. Cells were analyzed by flow cytometry and normalized to counting beads. (b) Representative Siglec-H staining (red) in PFA-fixated spleens of Wt and GM-CSF^-/-^ mice (right). The graph shows mean values ± SEM. *p-value<0.05, **p-value<0.01 compared with control group as determined by the Mann-Whitney-U-Test. Scale bars equal 400 μm.

GM-CSF is a pleiotropic cytokine that, in addition to its proinflammatory effects, also functions as a hematopoietic growth and differentiation factor. In this latter function, GM-CSF operates as an inhibitor of pDC development from DC precursors [17]. Because pDCs are critically involved in the pathogenesis of IMQPD [14], we investigated the impact of *Csf2* gene disruption on pDC differentiation, reasoning that the lack of GM-CSF could potentially be associated with a functionally relevant impairment in the negative regulation of pDC development. Comparative studies to determine the pDC content of cell preparations from the lymph nodes and spleens of healthy wild-type and GM-CSF^-/-^ mice in the absence of IMQPD were performed by flow cytometry analyses using Siglec-H expression as a pDC-specific marker. A significant increase in the pDC compartment within the CD45^+^ cell population was detected in the secondary lymphoid organs of GM-CSF^-/-^ mice (Fig 6a). These results were visualized by immunohistochemical analysis of Siglec-H^+^ cells in the spleens of GM-CSF^-/-^ compared with wild-type mice (Fig 6b). The red color of the alkaline phosphatase and the couterstaining with hematoxylin was too similar for distinction by an automated image anlysis tool for the quantification Siglec-H^+^ cells in the stained tissue specimens. Instead, we determined the numbers of Siglec-H^+^ cells in spleens of wild-type and GM-CSF-deficient mice by dividing stained images of the tissue into squares of equal size and counting the cells in a blinded fashion. The results of this semiquantitative analysis (data not shown) concurred with the visual impression of an increased content of Siglec-H^+^ cells in the spleens of GM-CSF-deficient compared with wild-type mice (Fig 6b) and agreed with results of the flow cytometry analysis (Fig 6a).

**Fig 7:**
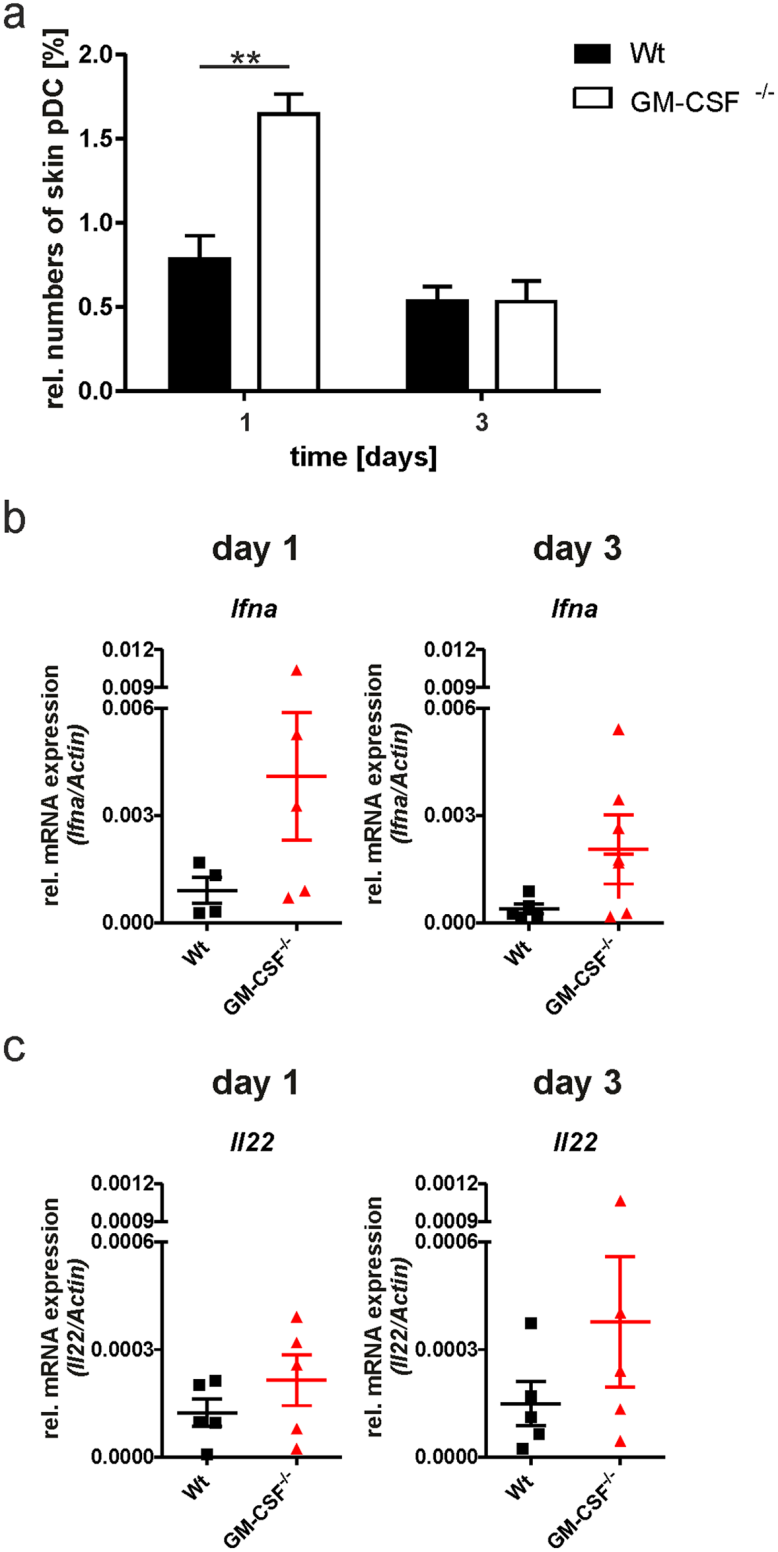
GM-CSF^-/-^ pDC are involved in the initial phase of IMQPD. (a) pDC distribution in skin of wild-type (Wt n = 5) and GM-CSF-deficient (GM-CSF^-/-^ n = 5) mice after 1 or 3 days of IMQ-induced psoriasis as analyzed by flow cytometry. (b) mRNA expression of IFNα (Ifna) in skin of wild-type and GM-CSF-deficient mice as analyzed by qPCR. (c) mRNA expression of IL22 (Il22) in skin of wild-type and GM-CSF-deficient mice as analyzed by qPCR. Bars in (a) show mean values ± SEM; for (b) and (c), symbols in graphs indicate mRNA levels in individual mice and horizontal bars show mean values ± SEM. **p-value< 0.01 compared with control group as determined by the Mann-Whitney-U-Test. Differences not marked by asterisks were not statistically significant.

As we detected an expansion of the pDC compartment in the spleen and lymph nodes of healthy GM-CSF-deficient mice without prior immune stimulation by IMQ, we were interested whether this subpopulation of myelomonocytic cells could contribute to IMQ-induced skin inflammation, thereby compensating for the lack of GM-CSF in GM-CSF^-/-^ mice. This cell population is a major source of IFN-α, especially during viral infection. As part of the innate immune response, pDCs are activated by recognition of endocytosed viral DNA via their toll-like receptors (TLR) to upregulate the expression of the proinflammatory cytokine IFN- α [18, 19]. In our experiments, IMQ, an agonist of TLR7 and known inducer of IFN- α secretion [20], was topically applied for the induction of IMQPD. As shown in Fig 7a, we found a significant increase in the percentage of Siglec-H^+^ pDC in the population of CD45^+^ cells in GM-CSF^-/-^ compared with wild-type mice at day 1 of IMQPD. Cutaneous IFN- α RNA expression levels were determined at days 1 and 3 of IMQPD to detect a signature for the functional contribution of skin-infiltrating pDC to the early development of skin inflammation (Fig 7b). IL-22 mRNA expression was assessed in parallel (Fig 7c), as pDCs have been demonstrated to promote T cell differentiation into an IL-22-expressing T helper cell phenotype (Th22) through the secretion of IL-6 and TNF-α [21]. Although IFN- α and IL-22 mRNA levels were numerically higher in GM-CSF^-/-^ compared with wild-type mice at days 1 and 3 after IMQPD induction but statistical significance was not achieved.

### Activated splenocytes derived from GM-CSF^-/-^ mice are prone to skewed IL-22 responses

**Fig 8:**
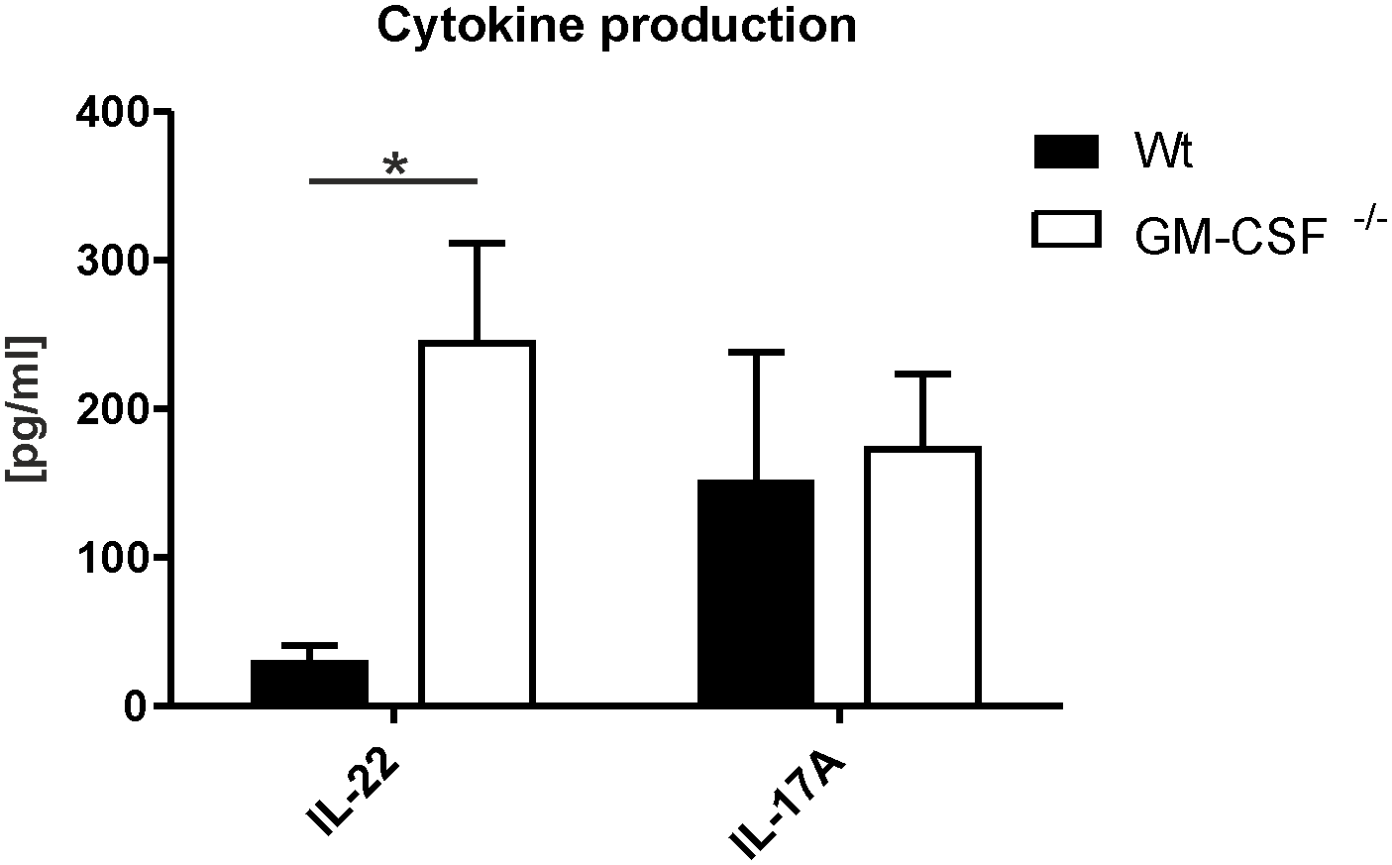
Cytokine levels in supernatants of GM-CSF-deficient T cells activated by costimulation. Isolated splenocytes of wild-type (Wt n = 5) and GM-CSF-deficient (GM-CSF^-/-^ n = 5) mice were activated by CD3 and CD28 stimulation, and concentrations of cytokines in supernatant were measured. IL-22 levels were determined by ELISA and IL-17A levels were measured by cytometric bead array. Bars show mean values ± SEM. *p-value<0.05, compared with control group as determined by the Mann-Whitney-U-Test.

The increased pDC cellularity in the skin of GM-CSF^-/-^ mice in the early phase of IMQPD development and its association with elevated IL-22 mRNA expression stimulated further investigations of the possibility that an inherited lack of GM-CSF might predispose mice to T cell responses with an IL-22- skewed effector cytokine pattern, based on published data on the pDC-mediated induction of IL-6- and TNFα-dependent differentiation of naïve T cells into effector cells producing IL-22 only [21]. Isolated splenocytes from wild-type and GM-CSF^-/-^ mice were activated by anti-CD3 and anti-CD28 costimulation *in vitro*, and the induced release of IL-17A and IL-22 was analyzed in the supernatants. Splenocytes derived from GM-CSF^-/-^ mice produced significantly increased levels of IL-22 upon activation compared with splenocytes from wild-type mice, whereas the release of IL-17A remained unchanged (Fig 8). Our investigations thus support the emergence of an IL-22-skewed cytokine pattern in effector T cells from mice genetically deficient for GM-CSF.

## Discussion

Recent progress in the understanding of pathogenic pathways driving psoriasis has highlighted the IL-23/Th17 axis as a critical component and led to the development of therapeutic strategies using cytokine-neutralizing antibodies to disrupt this pathway [2–5]. Among the downstream effector cytokines of this pathway, GM-CSF has been shown to contribute to the pathogenesis of other inflammatory diseases and is targeted in new therapeutic strategies for neuroinflammation [22] and rheumatoid arthritis [10, 11]. For our investigations on the potential of a recombinant anti-GM-CSF antibody to treat psoriasis, we chose the murine model of IMQPD because this complex model closely reflects several key histopathological and pathogenic features of human plaque psoriasis, thereby satisfying the requirements for a valid psoriasis model [23]. In particular, the IMQPD model is characterized by keratinocyte hyperproliferation and altered differentiation, papillomatosis, dermal infiltration with T cells, DC, monocytes, and neutrophils, a dependency of skin disease on T cell actions, and neoangiogenesis [14, 24]. Intriguingly, the model is also strongly dependent on the IL-23/IL-17 axis, which has been identified as a critical molecular pathway in human psoriasis and which has become the most effective therapeutic target in the treatment of this disease [1, 14, 25]. The most important limitation of the IMQPD model may be its lack of chronicity, another major feature of human psoriasis [23]; thus, it does not totally reflect the dynamics of psoriatic skin lesion development and its changes over time, but rather represents events that take place in early lesions [24].

In our study, GM-CSF inhibition by MOR012507 significantly ameliorated the severity of skin inflammation in the IMQPD model, lending additional support for the exploration of GM-CSF inhibition in the treatment of human plaque psoriasis in clinical trials. MOR012507 treatment decreased neutrophil infiltration and reduced expression of the proinflammatory cytokines TNFα, IL-1β, IL-6, and IL-22 in the skin. These alterations could potentially contribute to the therapeutic effect of MOR012507, as all of these cytokines have previously been implicated in the development of skin inflammation in the IMQPD model [26–29] and are also considered to play a role in human psoriasis [30]. Likewise, neutrophil depletion has previously been shown to attenuate the severity of skin inflammation in the IMQPD model [31]. The magnitude of this effect is similar to the reductions in skin inflammation we observed during GM-CSF inhibition, suggesting that the reduction in neutrophil recruitment by GM-CSF inhibition could contribute to its overall disease-ameliorating effect. Somewhat surprisingly, the functional role of neutrophils in human psoriasis has not been well studied despite its striking dominance at the histopathological level, specifically in early psoriatic plaques [30,32]. However, anecdotal studies report that psoriasis sometimes improves in psoriasis patients with neutropenia [33,34].

Although our initial findings suggested that the development of IMQPD involved GM-CSF as a critical component, subsequent investigations found an unaltered susceptibility of GM-CSF^-/-^ mice to IMQPD despite the absence of GM-CSF, thereby revealing an alternative pathogenic pathway. This alternative pathway does not seem to be readily activated in wild-type mice, but effectively compensates for the inherited lack of GM-CSF in mice with disruptions in the *Csf2* gene. We hypothesized that GM-CSF deficiency might be associated with an enhanced development of pDC. In support of this hypothesis, our studies demonstrated an expanded pDC compartment in secondary lymphoid tissues and increased pDC infiltration in the skin of GM-CSF-deficient wild-type mice. We also found an increase in IFNα mRNA expression as a pDC-specific cytokine signature in the skin of GM-CSF^-/-^ mice that was detectable within 1 day of IMQ induction; this increase was not observed in wild-type mice.

**Fig 9:**
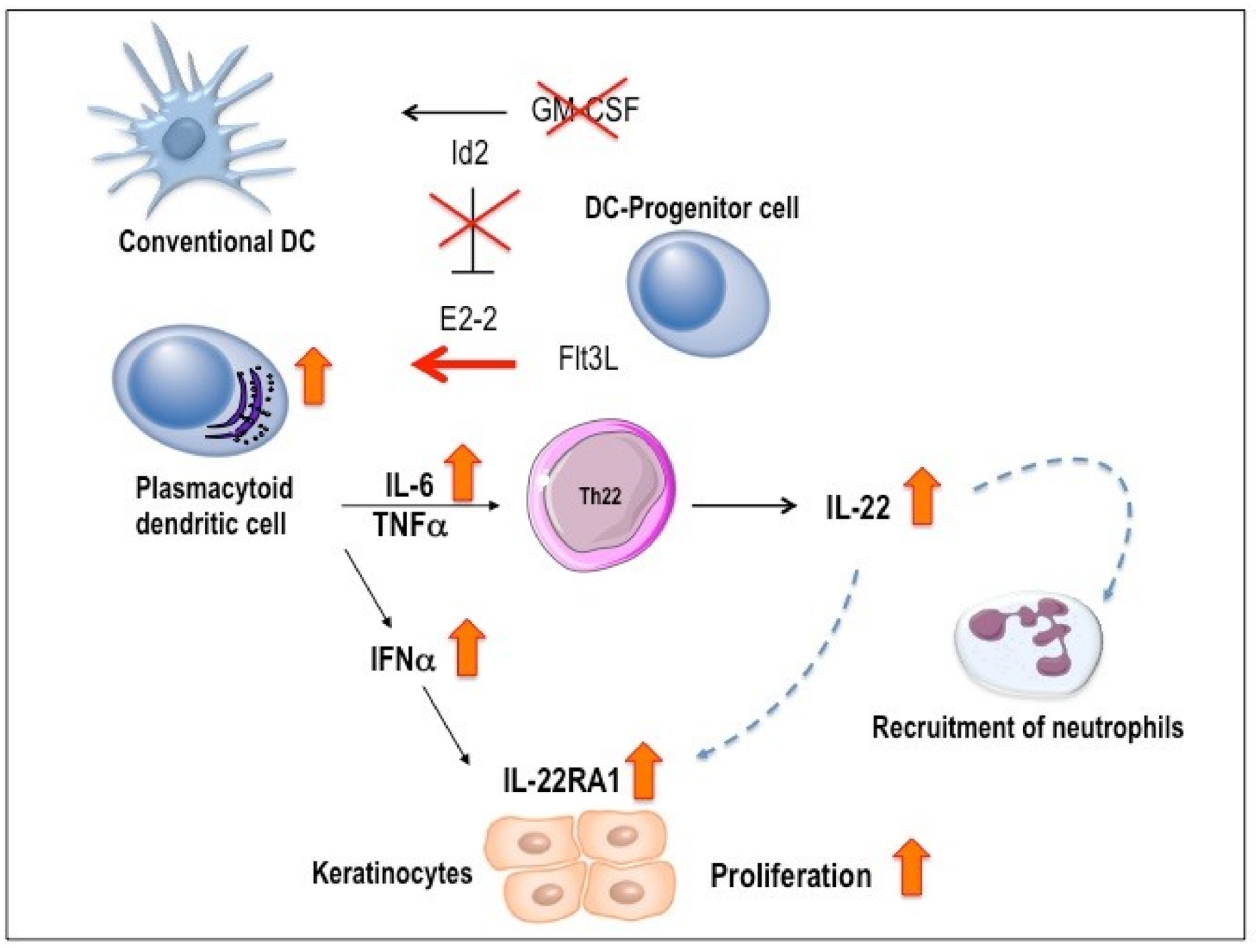
Hypothesis for the alternative pathogenic pathway of IMQ-induced skin disease in GM-CSF deficient mice. GM-CSF has a role as a differentiation factor in DC development; GM-CSF inhibits FLT3L-driven pDC production from lineage-negative DC precursor subsets, while promoting conventional DC growth via modulation of a complex network of transcriptional factors. Thus, the upregulation of the transcription factor E2-2, which is induced by FLT3L, is essential for the development and maintenance of a mature pDC phenotype. However, the E2-2-dependent pDC promoting-transcriptional program can be antagonized by GM-CSF via upregulation of the transcription factor ID2 that directs conventional DC development.

Our studies provide strong evidence that this alternative IMQPD pathway is associated with the role of GM-CSF as a differentiation factor in DC development [15]. In wild-type cells, GM-CSF is believed to inhibit FLT3L-driven pDC production from DC precursor subsets while promoting pDC growth via a complex network of transcription factors [35–37] (Fig 9). In cells with a genetic GM-CSF deficiency, this inhibitory FLT3L-driven pathway is lost, resulting in facilitated pDC development and an expanded pDC compartment. The significant increases in IL-22 and IL-6 expression observed in GM-CSF-deficient compared with wild-type mice on day 6 of IMQPD support our hypothesis of a functional role of the expanded pDC population in GM-CSF deficient mice during Th22 differentiation, while our investigations of IL-22 mRNA expression at earlier time points of IMQPD development provide insight into the sequence of events. Although the change in expression levels of IFN- α and IL-22 mRNA in GM-CSF-deficient compared with wild-type mice did not reach statistical significance at days 1 and 3 after IMQPD, the observed numerical variations are consistent with the hypothesis that the early significant increase in pDC skin infiltration at day 1 is accompanied by an intermittent rise in cutaneous IFN- α expression that is followed by an increase in cutaneous IL-22 expression between days 1 and 3, finally reaching statistical significance at day 6. This subsequently upregulated expression of IL-22 in the skin of GM-CSF^-/-^ compared with wild-type mice suggests a role for this cytokine in the alternative pathogenic pathway that compensates for GM-CSF deficiency. IL-22 is critical for development of IMQPD [27] and promotes keratinocyte differentiation, proliferation, and expression of proinflammatory and antimicrobial peptides [38–40].

Accordingly a genetic deficiency in GM-CSF might be associated with a facilitated pDC development resulting in an expanded pDC compartment, as demonstrated in our study which showed an enhanced pDC infiltration in secondary lymphoid tissues and in the skin of GM-CSF-deficient mice. The expanded pDC compartment leads to an upregulation of IFN-α as well as IL-6; in the presence of TNF-α, this pDC compartment contributes to the skewing of T cell responses towards an IL-22 dominated effector cytokine profile. IFN-α leads to an upregulation of IL-22 receptor mRNA expression in keratinocytes, thereby promoting the proliferative response of these cells to IL-22 stimuli. In addition, IL-22 induces an increase of skin infiltration by neutrophils. The vertical orange arrows in the figure indicate places where our experimental evidence supports the hypothetical scheme.

DC = dendritic cell; E2-2 = E box protein E2-2; FLT3L = FMS-like tyrosine kinase 3 ligand; GM-CSF = granulocyte-macrophage colony stimulating factor; ID2 = Inhibitor of DNA binding 2; IL-22RA1 = IL-22 receptor alpha chain; pDC = plasmacytoid DC

IL-22 appears to fulfill important physiological roles in wound healing and innate responses to protect against microbial attack at body barriers, where its receptor is mainly expressed by epithelial cells of the lung and gastrointestinal tract and by keratinocytes in the skin [41]. Like GM-CSF, IL-22 is capable of activating STAT3 and has been shown to upregulate the expression of proinflammatory and antimicrobial mediators such as S100 proteins and β-defensins in human keratinocytes [40,42]. In addition, IL-22 regulation of keratinocyte differentiation and proliferation and its potential as an inducer of epidermal hyperplasia have been demonstrated [38]. These biological activities are likely beneficial during wound healing and infectious processes, especially in conditions of a weakened innate immune response such as GM-CSF deficiency in genetically manipulated mice. Under these circumstances, IL-22 can provide a back-up cytokine defense at the outer and inner body surfaces, thereby protecting their structural and functional integrity. A weakened protective immunity and impaired wound healing are frequently occurring comorbidities in diabetes patients. Endogenous IL-22 is reduced at both the mRNA and protein level in diabetic inflammatory wounds, and topical IL-22 treatment results in accelerated diabetic wound closure by improving re-epithelialization [43]. However, these homeostatic effects are context dependent and closely connected with the potential of IL-22 to induce the expression of proinflamatory mediators by keratinocytes [40,42]. In response to a pathogenic trigger such as IMQ in IMQPD, IL-22 may act to help to fuel an inflammatory skin reaction rather than promote wound healing. Thus, the increased IL-22 expression upon IMQ challenge of GM-CSF-deficient mice might reflect a skewing of the T effector cell response toward a pro-pathogenic cytokine pattern. The skewed cytokine profile in GM-CSF-deficient mice, along with increased pDC cellularity, is reminiscent of conditions previously described for a TNFα- and IL-6-dependent pDC-induced pathway that promotes the differentiation of naïve T cells into an effector population expressing IL-22 only [21]. Our results with stimulated splenocytes from GM-CSF^-/-^ mice are consistent with the hypothesis that the inherited lack of GM-CSF provides a predisposing condition for the development of an IL-22-dominated T lymphocyte effector cytokine phenotype. Thus, enhanced IL-22 expression may compensate for the lack of GM-CSF in the recruitment of neutrophils to the skin in GM-CSF^-/-^ mice.

The alternative pathway revealed in our studies, which bypasses the inborn lack of GM-CSF in the pathogenesis of IMQPD, is not activated in wild-type mice during short-term antibody-induced GM-CSF blockade. Nor were there detectable changes in the pDC compartment when MOR012507 mAb was repetitively administered to wild-type mice for up to 4 weeks (results not shown), suggesting that GM-CSF blockade for longer periods of time may be required to induce such changes in the pDC lineage. However, it is possible that the use of long-term GM-CSF blockade as a therapeutic approach for humans with chronic inflammatory diseases could potentially trigger the described escape mechanisms, eventually leading to secondary treatment failure or adverse events. Aged GM-CSF^-/-^ mice are prone to developing a systemic lupus erythematosus-like disorder [44], a condition that is known to critically involve pDCs as a major source of the disease-promoting cytokine IFNα [45]. Accordingly, our results suggest that it would be prudent to monitor the pDC compartment and/or type I IFN signatures as biomarkers in patients treated with GM-CSF-neutralizing strategies to attenuate the potential risk of adverse effects. We emphasize, however, that the safety profile has been favourable during clinical development of GM-CSF blocking antibodies in rheumatoid arthritis, and the current clinical effectiveness data suggest that GM-CSF blockade is a valuable extension of available therapeutic options for inflammatory diseases [10, 11].

In conclusion, our investigations in the preclinical model of IMQPD provide strong evidence for the potential value of GM-CSF-neutralizing treatment in psoriasis. In the context of established treatment strategies for psoriatic skin disease, GM-CSF blockade might possess great potential, especially in the large subgroup of psoriasis patients with musculoskeletal involvement, due to its proven therapeutic benefit in inflammatory arthritis. The ongoing clinical development programs of this new treatment approach will provide further insights into its future place in the treatment armamentarium.

## Supporting information

S1 Table Fluorescently-labeled antibodies for flow cytometry. (DOCX)

S2 Table Primer sequences used for qPCR. (DOCX)
