# Supplementary material for: GM-CSF in murine psoriasiform dermatitis: Redundant and pathogenic roles uncovered by antibody-induced neutralization and genetic deficiency
Source: PLoS One. 2017 Aug 4;12(8):e0182646. doi: 10.1371/journal.pone.0182646 (PMC5544216; doi:10.1371/journal.pone.0182646)
Supplement: S1 Table — (DOCX) [file pone.0182646.s001.docx]

**S1 Table. Fluorescently-labeled antibodies for flow cytometry.**

| **Antibody** | **Clone** | **Manufacturer** |
| --- | --- | --- |
| CD3-PE-CF594 | 145-2C11 | BD Biosciences |
| CD11b-eFluor605 | M1/70 | Biolegends |
| CD11c-AlexaFluor700 | HL3 | BD Biosciences |
| CD45-VioBlue | 30-F11 | Miltenyi |
| Ly6C-PerCP.Cy5.5 | AL-21 | BD Biosciences |
| Ly6G-APC-Cy7 | 1A8 | Biolegends |
| MHCII-APC | M5/114.15.2 | Miltenyi |
| Siglec-H-FITC | 551 | Biolegends |
