# Supplementary material for: GM-CSF in murine psoriasiform dermatitis: Redundant and pathogenic roles uncovered by antibody-induced neutralization and genetic deficiency
Source: PLoS One. 2017 Aug 4;12(8):e0182646. doi: 10.1371/journal.pone.0182646 (PMC5544216; doi:10.1371/journal.pone.0182646)
Supplement: S2 Table — (DOCX) [file pone.0182646.s002.docx]

**S2 Table. Primer sequences used for qPCR.**

| **Gene** | **Forward** | **Reverse** |
| --- | --- | --- |
| *Actinb* NM_007393.5 | 5’-CCCTCGAACCCTAAGGCCA-3’ | 5’-GGGACACAACAGCCTGGATG-3’ |
| *Ifna* NM_008334.3, [NM_008333.2](https://www.ncbi.nlm.nih.gov/nucleotide/158631169?report=genbank&log$=nucltop&blast_rank=2&RID=BGBC5GD9013), [NM_010505.2](https://www.ncbi.nlm.nih.gov/nucleotide/158631167?report=genbank&log$=nucltop&blast_rank=3&RID=BGBC5GD9013), [NM_010503.2](https://www.ncbi.nlm.nih.gov/nucleotide/126722648?report=genbank&log$=nucltop&blast_rank=4&RID=BGBC5GD9013), [NM_010502.2](https://www.ncbi.nlm.nih.gov/nucleotide/117168292?report=genbank&log$=nucltop&blast_rank=5&RID=BGBC5GD9013), [NM_206975.1](https://www.ncbi.nlm.nih.gov/nucleotide/46195447?report=genbank&log$=nucltop&blast_rank=6&RID=BGBC5GD9013), [NM_206871.1](https://www.ncbi.nlm.nih.gov/nucleotide/46047422?report=genbank&log$=nucltop&blast_rank=7&RID=BGBC5GD9013), [NM_206867.1](https://www.ncbi.nlm.nih.gov/nucleotide/46047410?report=genbank&log$=nucltop&blast_rank=8&RID=BGBC5GD9013), [NM_206870.1](https://www.ncbi.nlm.nih.gov/nucleotide/46047407?report=genbank&log$=nucltop&blast_rank=9&RID=BGBC5GD9013), [NM_177347.2](https://www.ncbi.nlm.nih.gov/nucleotide/31340904?report=genbank&log$=nucltop&blast_rank=10&RID=BGBC5GD9013), [NM_177361.2](https://www.ncbi.nlm.nih.gov/nucleotide/31340762?report=genbank&log$=nucltop&blast_rank=11&RID=BGBC5GD9013), [NM_010507.1](https://www.ncbi.nlm.nih.gov/nucleotide/6754297?report=genbank&log$=nucltop&blast_rank=12&RID=BGBC5GD9013), [NM_010507.1](https://www.ncbi.nlm.nih.gov/nucleotide/6754297?report=genbank&log$=nucltop&blast_rank=12&RID=BGBC5GD9013), [NM_010504.2](https://www.ncbi.nlm.nih.gov/nucleotide/158631265?report=genbank&log$=nucltop&blast_rank=1&RID=BGBC8V2U016), [NM_008336.2](https://www.ncbi.nlm.nih.gov/nucleotide/113865992?report=genbank&log$=nucltop&blast_rank=11&RID=BGBC8V2U016) | 5’-CTTCCACAGGATCACTGTGTACCT-3’ | 5’-TTCTGCTCTGACCACCTCCC-3’ |
| *Il1b* NM_008361.4 | 5’-CAACCAACAAGTGATATTCTCCATG-3’ | 5’-GATCCACACTCTCCAGCTGCA-3’ |
| *Il6* NM_031168.2 | 5’-GAACAACGATGATGCACTTGC-3’ | 5’-TCTCTGAAGGACTCTGGCTTTG-3’ |
| *Il17* NM_010552.3 | 5’-CCACGTCACCCTGGACTCTC-3’ | 5’-CTCCGCATTGACACAGCG-3’ |
| *Il22ra1* NM_178257.2 | 5’-CTCACACCGGTCCTCTCG-3’ | 5’-TCTCTGTTTGCCTTCAAGGTG-3’ |
| *Ki67* NM_001081117.2 | 5’-GCTGTCCTCAAGACAATCATCA-3’ | 5’-GGCGTTATCCCAGGAGACT-3’ |
| *Tnfa* NM_013693.3 | 5’-GCCTCTTCTCATTCCTGCTTG-3’ | 5’-CTGATGAGAGGGAGGCCATT-3’ |

Primers for both *Csf2* (NM_009969) and *Il22* (NM_016971) were obtained from QIAGEN (Hilden, Germany).
